# Anthocyanin Accumulation and Molecular Analysis of Correlated Genes by Metabolomics and Transcriptomics in Sister Line Apple Cultivars

**DOI:** 10.3390/life12081246

**Published:** 2022-08-16

**Authors:** Caiyun Shi, Li Liu, Zhifeng Wei, Junwei Liu, Ming Li, Zhenli Yan, Dengtao Gao

**Affiliations:** Zhengzhou Fruit Research Institute, Chinese Academy of Agricultural Sciences, Zhengzhou 450009, China

**Keywords:** apple, anthocyanins, transcriptomics, transcription factor, WGCNA, MYB, bZIP

## Abstract

Red coloration in apples, an important quality trait, is primarily attributed to the accumulation of anthocyanins. Centuries of breeding have produced a wide variety of apples with different levels of anthocyanins in response to genetic and environmental stimuli. The Huashuo apple shows a much darker red color than its sister line, Huarui. Thirteen different anthocyanins were detected in Huashuo and Huarui apples, of which ten were significantly more abundant in Huashuo apples, confirming that the color difference is indeed attributed to high anthocyanins accumulation rather than the types of anthocyanins. In particular, the contents of cyanidin 3-*O*-galactoside levels were highest among anthocyanins in both cultivars, reaching >5000 μg·g^−1^ at the last color transition stage in Huashuo apples, while only >3000 μg·g^−1^ in Huarui apples. Moreover, the expression of most structural genes, especially *DFR*, *CHI*, and *4CL* associated with anthocyanin synthesis, were higher in Huashuo apples than in Huarui apples. Combined transcriptomics, metabolomics, and qRT-PCR analysis revealed that six transcription factors from the MYB and bZIP transcription factor families likely play key roles in the dark coloring of Huashuo apples. These results provide deeper insights into apple coloring and suggest a series of candidate genes for breeding anthocyanin-rich cultivars.

## 1. Introduction

Apple skin color, particularly anthocyanin-based coloration, is a key factor in fruit quality and market acceptance. Anthocyanins play several important roles in plants, including resistance to environmental stresses, protection against diseases, and pollination [1,2]. Additionally, anthocyanins have strong free radical scavenging and antioxidant capacity, which are important health-promoting properties [3], and are classified into six categories based on their molecular structures, namely malvidins, petunidins, pelargonidins, peonidins, delphinidins, and cyanidins [4].

Flowers with anthocyanin as the primary pigment show a wide range of colors, ranging from orange–red to red, purple, blue, and black. The red peel is mainly composed of three anthocyanins—cyanidin 3-*O*-galactoside, cyanidin 3-*O*-glucoside, and cyanidin 3-*O*-arabinoside—among which, cyanidin 3-*O*-galactoside is the primary component of the apple peel, while the other two are present in various amounts in different apple varieties [5]. In addition, apple peel contains small amounts of other anthocyanins, such as cyanidin 7-*O*-arabinoside, cyanidin 3-*O*-rhamnoside, and cyanidin 3-*O*-rutoside [5].

The anthocyanin synthesis pathway is a branch of phenylpropanoid biosynthesis [6] that involves a series of enzymes encoded by structural genes and is relatively conserved among plant species [7]. The structural genes affecting phenylpropanoid synthesis mainly include phenylalanine ammonia-lyase (*PAL*), cinnamic acid 4-hydroxylase (*C4H*), and 4-coumarate CoA ligase (*4CL*). The key branch point from monolignol biosynthesis to anthocyanins biosynthesis is catalyzed by chalcone synthase (*CHS*), chalcone isomerase (*CHI*), flavanone 3-hydroxylase (*F3H*), dihydroflavonol-4-reductase (*DFR*), anthocyanidin synthase (*ANS*), and UDP-glucose: flavonoid 3-*O*-glucosyltransferase (*UFGT*). *CHS*, *CHI*, and *F3H* are upstream structural genes, while *DFR*, *ANS*, and *UFGT* are downstream structural genes. The latter are usually key genes affecting anthocyanin synthesis, and their expression affects the anthocyanin content in tissues [8,9]. During apple fruit development, the co-expression of *CHS*, *F3H*, *DFR*, *ANS*, and *UFGT* is positively correlated with anthocyanin accumulation [10,11]. However, the expression patterns of these enzymes in apples differ among varieties, developmental stages, and tissues [12].

Transcription factors (TFs) are involved in the color regulation of plant organs. Anthocyanin biosynthesis is regulated by the MYB-bHLH-WD40 (MBW) protein complex [13,14]. MYB is a well-studied TF family in plants and classified into four categories based on the number of domains: 1R-MYB, R2R3-MYB, 3R-MYB, and 4R-MYB, of which R2R3-MYB is the most abundant [9,15,16,17]. Several MYB family TFs involved in anthocyanin synthesis have been identified in apples, including *MdMYB1* [11], *MdMYBA* [18], *MdMYB10* [19], *MdMYB110a* [20], *MdMYB*3 [21], *MdMYB9*, *MdMYB11* [22], and *MdMYB90-like* [23]. MYB TFs negatively regulating anthocyanin synthesis have also been reported. For example, the anthocyanin content in red apple calli is significantly reduced if *MdMYB16* [24] or *MdMYB111* [25] is overexpressed. A recent study found that *MdMYB306-like* inhibited the anthocyanins synthesis in apple peels by interacting with *MdMYB17* and *MdbHLH33* [26]. Studies have identified more TFs in anthocyanin accumulation, such as B-box protein [27,28,29,30], NAC [31], WRKY [32,33], and bZIP [34,35].

High-throughput methods, such as transcriptomic and metabolomic approaches, have been widely used to explore coloring-related mechanisms in fruits, including apples [36,37,38]. The Huashuo apple is a hybrid of Huaguan and American No.8 apples cultivated by the Zhengzhou Fruit Research Institute, Chinese Academy of Agricultural Sciences [39]. Huashuo apples have been cultivated in China. The Huashuo apple has a deeper red color than its sister line, Huarui, with both planted in Zhaotong City (Yunnan Province, China). This study determines why the coloring of the Huashuo apple differs from that of the Huarui apples using transcriptome and metabolome analysis. The mechanism underlying the Huashuo-specific coloration may provide a theoretical basis and gene resources for the breeding of apple color-dominant traits, which may appeal to consumers, thereby enhancing market value.

## 2. Materials and Methods

### 2.1. Plant Materials

Two apple cultivars, Huashuo (YS) and Huarui (YR), were planted in the orchard located at Zhaotong Apple Industry Research Institute (Zhaotong, China). The trees were five years old, planted at 3.5 × 1.5 m intervals between and along rows, and grafted onto M26 interstock. Nine trees with consistent and healthy growth were selected for sampling from each cultivar, with three trees selected as biological replicates. Samples were collected over the color transition period of 105, 110, 120, and 130 days after full blossom (DAFB); similarly, 15 fruits were collected from the three biological replicate. After separating the peel from the cortex (1 mm thick), some of the mixed peel samples were collected for pigment measurement while the remaining samples were frozen in liquid nitrogen and stored at −80 °C for subsequent metabolome and transcriptome analyses.

### 2.2. Measurement of Apple Skin Color Traits

#### 2.2.1. Relative Total Anthocyanin Content

Anthocyanin was determined as previously reported, with modifications [40,41]. Apple peel samples (5 g) were crushed into powder and extracted with 20 mL of 1% HCl in methanol (*v*/*v*) for 24 h at 4 °C in the dark. Subsequently, the mixture was filtered, and absorbance of the filtrate was measured at 530 nm using an ultraviolet spectrophotometer (Shimadzu UV-2450, Kyoto, Japan). The relative anthocyanin concentration was calculated using the following formula:

N = 10 × A_530nm_/m

where A_530nm_ refers to the absorbance value of the sample at 530 nm, m represents the mass of the sample, and N is the relative anthocyanin concentration of per gram fresh weight (Uint/g FW).

#### 2.2.2. Measurement of Anthocyanin Metabolites by HPLC-ESI-MS/MS

Anthocyanin metabolites were obtained from Metware Biotechnology Co., Ltd. (Wuhan, China). Apple peel samples were freeze-dried in a vacuum freeze dryer (LGJ-22, Sihuan Tech Co., Beijing, China) for 64 h in the vacuum environment as the temperature gradually rose from −50 °C to 30 °C, and crushed using an MM 400 mixer mill (Retsch Co., Haan, Germany) and zirconia beads for 1.5 min at 30 Hz. The crushed samples (50 mg) were mixed with 0.5 mL of 50% aqueous methanol (including 0.1% HCL, *v*/*v*), vortexed for 5 min, ultrasonicated for 5 min, centrifuged for 5 min, and the supernatants were isolated; this process was repeated twice. The combined supernatants were filtered through a SCAA-104 membrane (0.22 μm pore size; ANPEL, Shanghai, China) and analyzed using a UPLC-ESI-MS/MS system (UPLC, ExionLC™ AD, https://sciex.com.cn/); MS, Applied Biosystems 6500 Triple Quadrupole, https://sciex.com.cn/). The analytical conditions were as follows: UPLC: column, WatersACQUITY BEH C18 (1.7 µm, 2.1 mm × 100 mm); solvent system, water (0.1% formic acid), methanol (0.1% formic acid); gradient program, 95:5 (*v*/*v*) at 0 min, 50:50 (*v*/*v*) at 6 min, 5: 95 *v*/*v* at 12 min, hold for 2 min, 95:5 (*v*/*v*) at 14 min, hold for 2 min; flow rate, 0.35 mL/min; temperature, 40 °C; injection volume, 2 μL. ESI-MS/MS: ion source, ESI, source temperature 550 °C; ion spray voltage (IS) 5500 V; curtain gas (CUR) was set at 35 psi. In Q-Trap 6500+, each ion pair was scanned and detected according to the optimized declustering potential (DP) and collision energy (CE).

### 2.3. RNA Sequencing and Analysis

Total RNA from each peel sample was extracted using a Column Plant RNA Extraction Kit (Sangon Biotech Co., Shanghai, China). The quality and concentration of the RNA samples were determined using an Agilent 2100 Bioanalyser (Agilent Technologies, Santa Clara, CA, USA), Qubit 3.0 fluorometer (Invitrogen, Waltham, MA, USA), and NanoDrop 2000 spectrophotometer (Thermo Fisher Scientific, Waltham, MA, USA). High-quality total RNA (1 μg) was used to synthesize first-strand cDNA with the Prime Script RT Reagent Kit with gDNA Eraser (TaKaRa Bio, Dalian, China).

The raw RNA-Seq data were cleaned by removing adaptor sequences and low-quality reads using Cutadapt v1.18 and Trimmomatic-0.38 software, respectively [42,43]. The clean reads were mapped to the Malus domestic v1.1 genome (https://phytozome-next.jgi.doe.gov/, accessed on 25 November 2021) using the hisat2 software for assembly. Gene expression levels were estimated using HTseq [44]. Pearson correlation coefficients between biological replicates were calculated using the corrplot package in R [45].

### 2.4. WGCNA and Visualization of Gene Networks

Weighted gene co-expression network analysis (WGCNA) [46] was performed (soft threshold for 16, minimum module size for 100, and combined cutting height for 0.3) on the gene expression profile, including 10 different anthocyanins and the total anthocyanin content. Correlation analysis was conducted between the identified modules and the total anthocyanin metabolism in the peel. Modules with the highest correlation were selected based on the Pearson correlation coefficient (PCC).

### 2.5. Quantitative Real-Time PCR (qRT-PCR) Validation of RNA-Seq Results

Six genes were selected for qRT-PCR analysis to examine the different expression profiles. Methods for RNA extraction and cDNA synthesis are provided in Section 2.3. qRT-PCR was performed in a 10 μL reaction volume using SYBR Premix Ex Taq (TaKaRa, Dalian, China) on a Quant Studio 7 Flex Real-Time PCR System (Applied Biosystems, Foster City, CA, USA) according to the manufacturer’s protocol. The primers were designed using Primer 5.0 software; sequences are listed in Table S1. The primers for actin served as the internal control: forward primer, 5′-TGACCGAATGAGCAAGGAAATTACT-3′, and reverse primer, 5′-TACTCAGCTTTGGCAATCCACATC-3′. qRT-PCR was conducted with three biological replicates, all of which had three technical replicates. The reaction conditions were as follows: initial incubation at 50 °C for 2 min, followed by 95 °C for 2 min, and 38 cycles of 95 °C for 15 s, 60 °C for 15 s, and 72 °C for 20 s. The relative expression of the targeted genes was calculated using the negative delta-delta Ct method [47].

### 2.6. Function Prediction of Candidate Genes

The target protein sequence was uploaded to the online server STRING to predict the interaction network (https://cn.string-db.org/cgi/input.pl, accessed on 20 March 2022) with an option value of >0.700 [48,49,50]. The search box selected was the single protein by sequence; the organism was Malus domestica; the max number of interactors to show no more than 10.

### 2.7. Statistical Analyses

To visualize specific relationships, correlation network analysis was performed using the online server OmicStudio tools (https://www.omicstudio.cn/tool, accessed on 26 April 2022). The data were analyzed with Excel 2010 (Microsoft Corp., Redmond, WA, USA); Sigmaplot v14.0 (Systat Software, San Jose, CA, USA) software was used to create column and line charts. Significant differences in anthocyanin contents or gene expression levels in different samples were determined using Duncan’s multiple range test (*p* < 0.05) in the ANOVA (analysis of variance) program of SPSS 19.0 (SPSS Inc., Chicago, IL, USA).

## 3. Results

### 3.1. Differences in Anthocyanin Types and Total Content between Apple Cultivars

**Figure 1:**
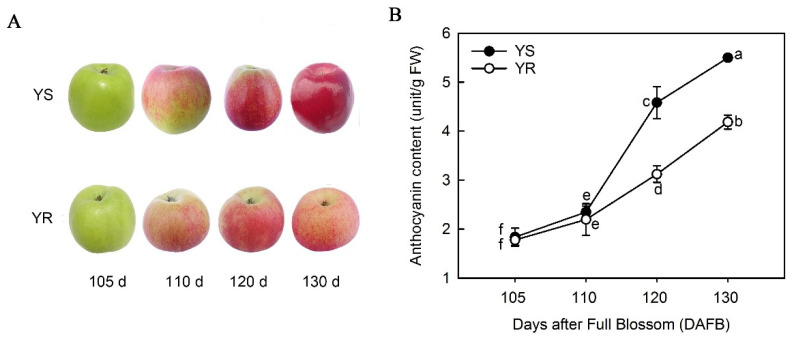
Changes in appearance and anthocyanin content during the color transition period. (**A**) Apple phenotypes. (**B**) Total anthocyanin content. Lowercase letters indicate significant differences at *p* < 0.05 using Duncan’s multiple range test. YS, Huashuo; YR, Huarui.

The total anthocyanin content of YS and YR was determined using ultraviolet spectrophotometry. Figure 1A illustrates the phenotype of YS and YR, showing different degrees of coloration over the color transition period; 130 DAFB was the commercial maturation stage of YS. The entire surface of YS was a strong red color at 130 DAFB, while YR was much lighter in color. The total anthocyanin content gradually increased over time, and was significantly higher in YS than YR at 120 and 130 DAFB (Figure 1B).

**Figure 2:**
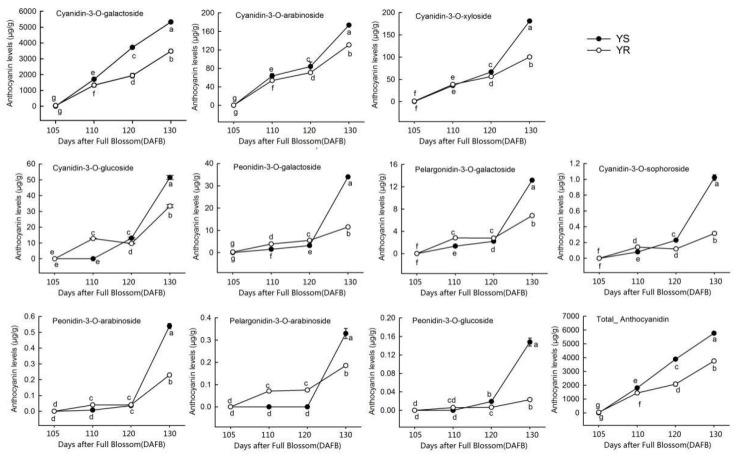
Anthocyanin levels during the color transition period. Different lowercase letters indicate significant differences at *p* < 0.05 using Duncan’s multiple range test. YS, Huashuo; YR, Huarui.

A total of 13 different anthocyanins were detected using HPLC-ESI-MS/MS, of which six were cyanidins, three were peonidins, two were pelargonidins, and the remaining two were delphinidins (Table S2). Changes in anthocyanin content were not consistent throughout the color transition period. Levels of cyanidin-3-*O*-rutinoside and delphinidin-3-*O*-arabinoside did not increase gradually during the color transition period, while delphinidin-3-*O*-glucoside was only detected in the last color transition stage of YR. Therefore, the 10 gradually accumulated anthocyanins were selected for analysis (Figure 2). Cyanidin-3-*O*-galactoside showed the highest level among all anthocyanins in both cultivars, reaching >5000 μg·g^−1^ g at 130 DAFB in YS, followed by cyanidin-3-*O*-arabinoside and cyanidin-3-*O*-xyloside. In the early color transition stages, some anthocyanins had higher levels in YR than in YS; however, most anthocyanins were significantly less abundant in YR than in YS at 130 DAFB (Figure 2).

### 3.2. Transcriptome Sequencing and Analysis

Four sampling time points underwent transcriptome sequencing for each cultivar. Sample quality control data are shown in Table S3, including the total base count, average read length, Q20 base ratio, Q30 base ratio, and GC base ratio. Among these, the Q20 base ratio was over 97%, while the Q30 base ratio was >90%. In each sample, most genes were between 1–10 bp and 10–100 bp in length, with fewer genes between 0.5–1 bp or >100 bp in length (Figure S1). The average gene expression level in YS and YR fluctuated slightly, with the gene expression in HS showing a downward trend. Meanwhile, the gene expression in HR showed larger differences in average expression level (Figure S1). To further confirm the RNA-Seq results, qRT-PCR analysis was performed on randomly selected genes (Figure S2). It can be seen that the FPKM (Fragments Per Kilobase of exon model per Million mapped fragments) values by RNA-seq were consistent with the transcript levels by qRT-PCR, suggesting that the sequencing data are reliable.

### 3.3. WGCNA Analysis of Anthocyanin Contents and Transcriptome Data

**Figure 3:**
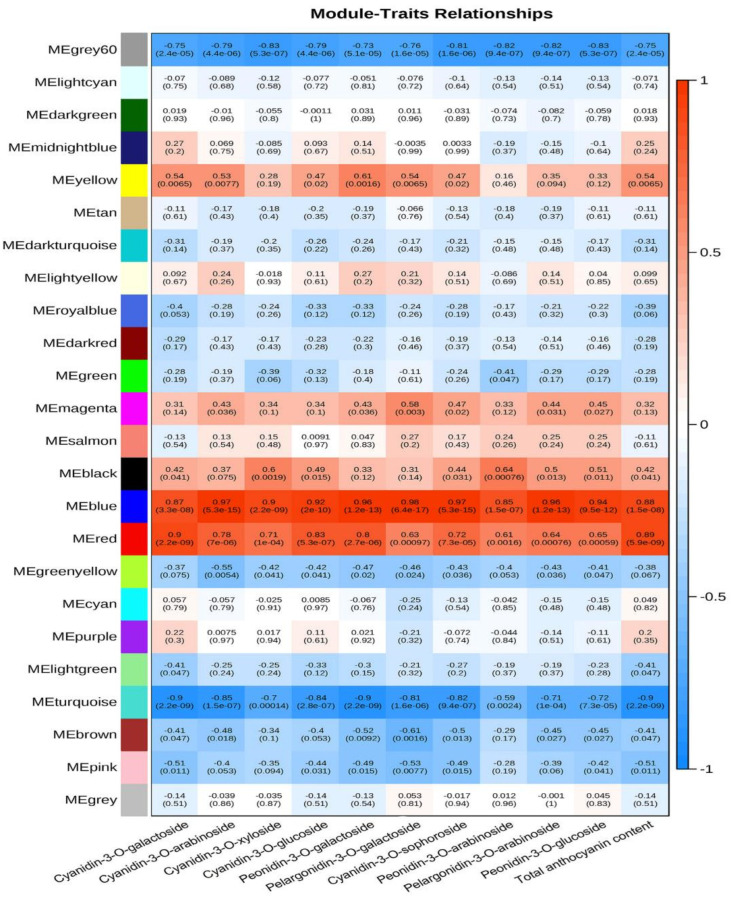
Association analysis of transcriptome and metabolome. Different module colors represent the correlation between the genes of the module and anthocyanins. The brighter the red (the value is closer to 1), the higher the positive correlation, while the brighter the blue (the value is closer to −1), the higher the negative correlation. The number in brackets represents significance (*p* value), with the smaller the value, the stronger the significance. The horizontal axis is the type of anthocyanins.

**Figure 4:**
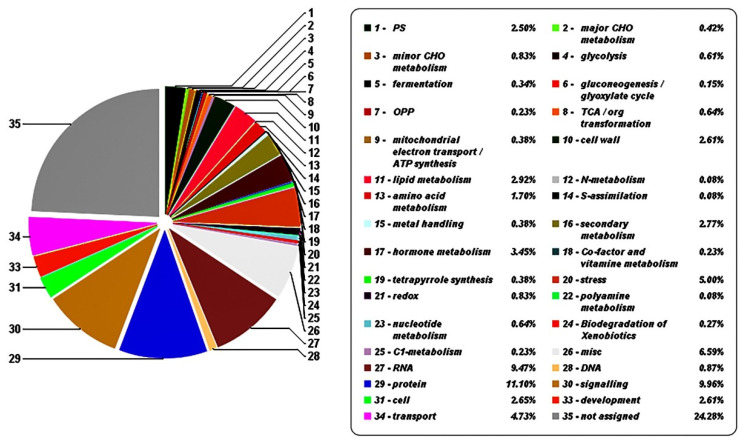
Functional annotation of genes with correlation value of ≥0.8 or ≤−0.8. The left side shows the screened genes divided into different categories; the right side introduces the name of each part and their proportions in detail.

Ten different anthocyanins and total anthocyanin content underwent WGCNA analysis (Figure 3). Different modules represent a class of genes whose transcript levels were consistent with the accumulation of this type of anthocyanins. As shown in Figure 3, for the color of each module, the brighter the red, the higher positive correlation, while the brighter the blue, the higher negative correlation. Highly positive correlated genes were mainly distributed in the blue and red modules, while highly negative correlated genes were mainly distributed in the grey60 and turquoise modules. The screened genes based on the correlation value of ≥0.8 or ≤−0.8 were annotated using the plaBi database. Figure 4 shows the proportions of different gene categories. All genes were divided into 35 subclasses. Functions associated with signaling, RNA, protein, and stress transport accounted for a relatively high proportion. The 16th subclass was secondary metabolites, accounting for 2.77%, while the 17th subclass was hormone metabolism, accounting for 3.45%. TFs were in the 27th subclass, accounting for 9.47%.

### 3.4. Expression of Genes Associated with Regulation of Anthocyanin and Phenylpropanoid Metabolism

**Figure 5:**
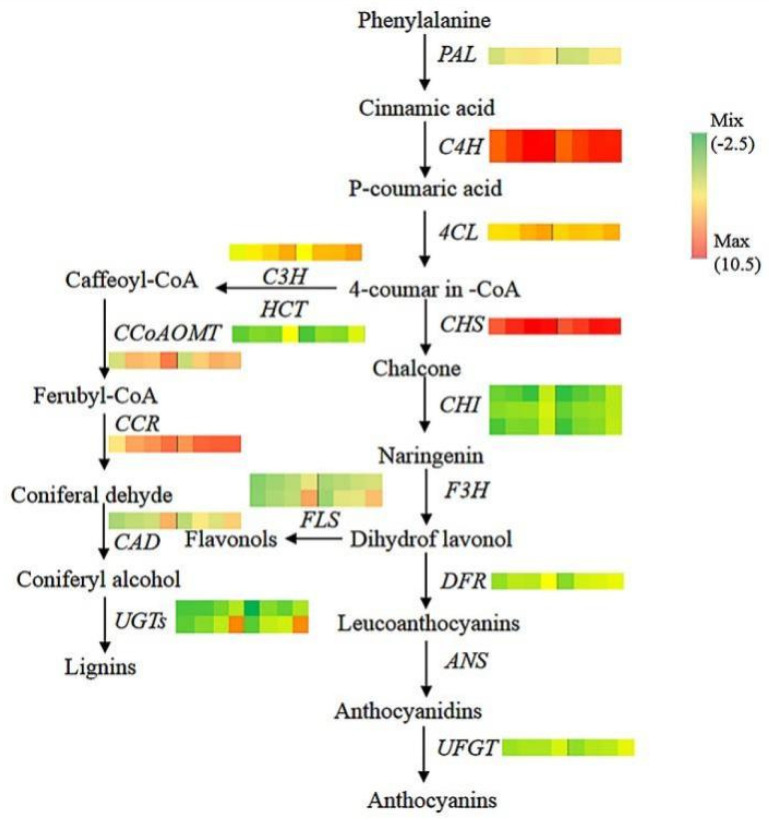
Overview of modulation of the phenylpropanoid pathway. The pathway was adapted from Ji et al. [51]. For each gene, the eight squares from left to right represent YS1–4 and YR 1–4. The number of rows represents the number of transcripts. Different colors represent a downregulated or upregulated gene. YS, Huashuo; YR, Huarui; *PAL*, phenylalanine ammonia lyase; *C4H*, cinnamate-4-hydroxylase; *4CL*, 4-coumaroyl-coA synthase; *CHS*, chalcone synthase; *CHI*, chalcone-flavanone isomerase; *F3H*, flavanone-3-hydroxylase; *FLS*, flavonol synthase; *DFR*, dihydroflavonol-4-reductas; *UFGT*, UDP-glucose: flavonoid-3-*O*-glucosyltransferase; *CCoAOMT*, caffeoyl CoA o-methyl transferase; CCR, caffeoyl CoA reductase; *CAD*, cinnamoyl alcohol dehydrogenase; *UGT*, UDP-glycosyltransferase; *C3H*, p-coumarate 3-hydroxylase; *HCT*, hydroxycinnamoyl-coA shikimate/quinate hydroxycinnamoyl transferase.

Several genes from the 16th subclass (Figure 4) were associated with anthocyanin and lignin synthesis, both of which belong to the phenylpropanoid pathway. These genes are schematically represented in Figure 5. Transcript levels of these genes gradually increased throughout the color transition period. During the four sampling time points, transcript levels of structural genes upstream of anthocyanin synthesis pathway, including *PAL*, *C4H*, *CHS*, *CHI*, and *DFR*, were higher in YS than in YR. The expression trends of *PAL*, *CHI*, and *DFR* determined by qRT-PCR were consistent with those of the transcriptome data (Figure S2). Moreover, the transcript levels of genes associated with flavonol and lignin synthesis, such as *FLS* (flavonol synthase), *CCoAOMT* (caffeoyl CoA O-methyltransferase), *CCR* (caffeoyl CoA reductase), *C3H* (p-coumarate 3-hydroxylase), *HCT* (hydroxycinnamoyl-coA shikimate/quinate hydroxycinnamoyl transferase), and *CAD* (cinnamoyl alcohol dehydrogenase), also gradually increased as the color deepened. *C4H* had two copies with similar expression levels over the entire color transition period; *CHI* had three copies, one of which had significantly higher levels than the other two during the entire color transition period. FLS had two copies with inconsistent expression levels, although the expression levels of these copies gradually increased throughout the color transition period. Regarding genes associated with lignin synthesis, the expression levels of *HCT*, *CCoAOMT*, and *CAD* in YS were much higher than those in YR.

Furthermore, some hormone-related genes may also play key roles in anthocyanin accumulation. WGCNA revealed that the absolute value of the correlations between these genes and anthocyanin levels reached >0.8 (Table S4). Among them, the transcripts of CTK (cytokinin), JA (jasmonic acid), ETH (ethylene), and SA (salicylic acid) were positively correlated with the anthocyanin content. Meanwhile, ABA (abscisic acid) had one transcript that was negatively correlated with anthocyanin accumulation, while BR (brassinolide) and GA (gibberellic acid) had two. Among the 12 transcripts of auxin, 7 were positively correlated with anthocyanin content and 5 were negatively correlated (Table S4).

### 3.5. Key Transcription Factors and Structure Genes Associated with Anthocyanin Accumulation and Their Interaction Network

**Figure 6:**
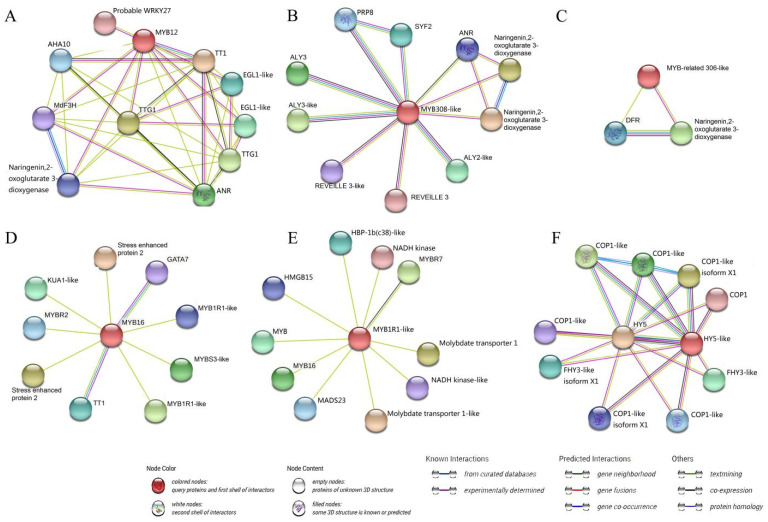
Interaction network analysis for key transcription factors. (**A**–**F**) sequentially represent the interaction network of six transcription factors (MYB12, MYB308-like, MYB-related 306-like, MYB16, MYB1R1-like, HY5-like) we screened. Colored nodes represent different proteins; the red is the target protein I upload. Node content: empty nodes represent proteins with unknown 3D structure, while filled nodes represent proteins with known or predicted 3D structure. Lines of different colors in the middle of nodes represent different relationships of proteins.

Several AP2/EREBP, ARF, C2C2, C2H2, HB, bHLH, MYB, and WRKY family TFs correlated with anthocyanin accumulation (Table S5), six of which were predicted to interact with anthocyanin-related proteins using the STRING server (Figure 6). Among them, five TFs belonged to the MYB family, while one belonged to the bZIP family. MDP0000140609 was most likely MYB12 and annotated as an anthocyanin regulatory protein, and the interactions with F3H, TT1 (transparent testa 1), and TTG1 (transparent testa glabra1-like, a WD40 protein) were experimentally determined, while the interactions with ANR (anthocyanidin reductase, catalyze anthocyanidin to form proanthocyanidin) was predicted (Figure 6A). MDP0000250597 was annotated as MYB308-like protein and could interact with ANR, naringenin,2-oxoglutarate 3-dioxygenase, ALY (always early) proteins, PRP8 (pre-mRNA-processing-splicing factor 8), REVEILLE3, REVEILLE3-like, and SYF2 (pre-mRNA-splicing factor 2) (Figure 6B). MDP0000284922 was predicted to be similar to the MYB-related 306-like protein in the string database, while the annotation in the plaBi database was putative MYB109 protein; this was predicted to interact with DFR and naringenin,2-oxoglutarate 3-dioxygenase, but the interaction with DFR was not verified (Figure 6C). MYB16 (MDP0000950559) was involved in the response to UV-B according to the gene annotation, and interacted with TT1 and GATA7, and also was predicted to interact with other proteins such as MYBs, KUA1 (KUODA1)-like, and stress-enhanced protein 2 (Figure 6D). MDP0000074681 was annotated as MYB-related TF family, possibly MYB1R1-like protein, and predicted to interact with MYB16, MADS23 (MADS-box transcription factor 23), molybdate transporter 1, molybdate transporter 1-like, NADH kinase, NADH kinase-like, MYBR7, HMGB15 (high mobility group B protein 15), HBP-1B(c38)-like (hex-motif-specific DNA-binding protein 1B subfamily c38), and another unnamed MYB (Figure 6E). The HY5-like (MDP0000834642), which belongs to the bZIP family, was predicted to interact with HY5, which integrates light and hormone signaling pathways, and several COP1 (constitutive photomorphogenesis protein 1), COP1-like, FHY3 (far-red elongated hypocotyl 3)-like, and FHY3-like isoform proteins (Figure 6F).

According to the prediction results of the above key TFs, some TFs can interact with F3H or DFR which are directly involved in anthocyanin biosynthesis. In addition, we selected other genes, 4CL and CHI, which the transcription levels in the whole color transient period in YS were much higher than that in YR, for interaction analysis. Since the correlation between the dynamic accumulation of anthocyanin and the transcription level of F3H was <0.8, we only selected DFR, 4CL, and CHI for protein interaction prediction (Figure S3). DFR (MDP0000607969) can interact with FLS, ANS, F3H, and other proteins related to anthocyanin biosynthesis (Figure S3A). The 4CL (MDP0000691789) can interact with proteins of anthocyanin synthesis (F3H, PAL, and probable CHI3) and lignin synthesis (CCoAOMT, CCR1-like) related, along with some other enzymes (naringenin,2-oxoglutarate 3-dioxygenase and feruloyl CoA ortho-hydroxylase 1-like) (Figure S3B). The CHI (MDP0000205890) can interact with 4CL, ANS, F3H, CHI isoform, anthocyanin synthesis-related proteins, and other enzymes.

## 4. Discussion

The anthocyanin biosynthesis pathway is a well-studied secondary metabolite pathway in plants [52]. The mechanism of action of structural genes has been well described, while several TFs involved in the regulation of anthocyanin biosynthesis have been identified. Herein, we have identified the material basis for the coloration differences between sister lines Huashuo and Huarui, discovered associated key TFs, and analyzed the expression of structural genes over the entire color transition period for both cultivars.

Anthocyanin accumulation is responsible for the apple skin’s observed red color [53]. Cyanidin-3-*O*-galactoside, the primary anthocyanin in apple peel, accounts for >85% of the total anthocyanin content. Other anthocyanins, such as cyanidin 3-*O*-arabinoside and cyanidin 3-*O*-glucoside have also been identified in apple peel [53,54]. Tsao et al. [55] suggested that cyanidin 3-*O*-galactoside is unique and only found in red apple peels. In the current study, the anthocyanin content increased as the peel’s red color gradually deepened. Moreover, cyanidin 3-*O*-galactoside levels were highest among anthocyanins in both YS and YR (Figure 2), which is consistent with previous findings [53,56]. The difference in peel coloring between the two cultivars was primary attributed to the high accumulation of anthocyanins, not differences in anthocyanin type, since a gradual increase in anthocyanins levels was detected in both cultivars. Moreover, some anthocyanins were not detected in the early color transition stages for either YS or YR, possibly due to their low concentrations.

Anthocyanins are synthesized via the phenylpropanoid pathway. Phenylpropanoid metabolism produces structurally diverse plant secondary metabolites such as anthocyanins, flavonoids, lignins, and proanthocyanidins [57]. Anthocyanin biosynthesis is related to the key structural genes involved in flavonoid and anthocyanin biosynthesis, such as *PAL*, *CHS*, *CHI*, *F3H*, *DFR*, *ANS*, and *UFGT* [56]. *PAL* is a key rate-limiting enzyme in the flavonoid pathway located upstream of the anthocyanin synthesis pathway [58]. In this study, the expression of *PAL* in YS and YR gradually increased during the color transition period; at each time point, *PAL* expression in YS was higher than that in YR, which might have accelerated anthocyanin accumulation in YS (Figure 5). *C4H* expression remained at a high level in both cultivars, even in the first color transition stage. Previous studies have reported that anthocyanin production in plants does not always correlate with the transcriptional activity of the first two genes, *CHS* and *CHI* [59,60]; our findings indicated a high correlation between transcript levels of these genes with anthocyanins accumulation. Although not every transcript had the same expression level, one of these genes might have played a major role in anthocyanin accumulation (Figure 5). Kim et al. [61] isolated cDNA clones for *F3H*, *DFR*, *ANS*, and *UFGT* from apple skins, and reported preferential expression in apple skin tissue, especially red skin, which was light induced. In the current study, DFR expression in YS was significantly higher than that in YR, while the correlation with anthocyanin content reached 0.91, suggesting that DFR might be the key gene promoting anthocyanin accumulation.

Specific genes involved in the lignin and flavonoid biosynthesis pathways were also identified in the current study. The expression of these genes gradually increased as the fruit ripened and red color deepened. Anthocyanin and lignin biosynthesis are metabolically interconnected and diverged in their central metabolite, 4-coumaroyl CoA. *C4H* is known to play a key role in lignin biosynthesis [62]. Ring et al. [63] suggested that the downregulation of *CHS* and concomitant induction of *FaPRX27* expression diverted the flux from anthocyanins to lignin. *CHS* expression was much higher than that of genes associated with the lignin and flavonol synthesis pathway genes during the color transition period in YS and YR, indicating that many substrates are involved in the synthesis of anthocyanins. These results highlight the competition between the different phenolic pathways for their common precursors.

Hormones can regulate anthocyanin formation [52]. Endogenous ABA and ETH are important inducing factors for the accumulation and synthesis of anthocyanin. Further, IAA (indole-3-acetic acid) has been shown to restrain the accumulation of sugars and anthocyanin synthesis in blueberries, although the regulatory function of GA3 remains unclear [64]. Li et al. [65] suggested that IAA had a positive regulatory effect on the synthesis of anthocyanins in apples, whereas GA had the opposite effect. A previous study also showed that application of ETH to strawberries at the immature stage repressed anthocyanin biosynthesis [66]. These findings demonstrate that hormones may play different roles in different maturation stages of different species. In the current study, various hormones were strongly correlated with anthocyanin accumulation (Table S4), suggesting that different transcripts of the same hormone may promote or inhibit anthocyanin accumulation through various mechanisms, such as interaction with MYB, bZIP, or other TFs.

In addition to its key structural genes, anthocyanin biosynthesis is regulated by several TFs, such as members of the MYB, MADS-box, bHLH, WD40, bZIP, and WRKY families [67,68]. Numerous TFs, including AP2/EREBP, MYB, bHLH, C2C2, WRKY, C2H2, HAP, HB, and AS2 families, were identified in the correlation analysis between the transcriptome and anthocyanin metabolome (Table S5), of which only six were predicted to be related to anthocyanin accumulation. Studying interaction networks contributes to a better understanding of protein biological functions and molecular processes. The anthocyanin type, correlation value, and positive or negative correlation with specific TFs are shown in Figure S4. Five MYBs and one bZIP TF were predicted to interact with anthocyanin-related proteins such as F3H, DFR, 4CL, TT1, TTG, and other TFs or intermediate enzymes (Figure 6). The *TT1* gene encodes a WIP-type zinc finger protein and is required for correct expression of proanthocyanidin-specific genes in the seed coat of *Arabidopsis thaliana*, but also affects *CHS*, a key gene related to anthocyanin synthesis [69]. The *TTG1* is a WD40 protein and controls the biosynthesis of anthocyanins and proanthocyanidins by interacting with bHLH proteins [70]. Among the five MYB TFs, MYB12 (MDP0000140609) showed interaction with F3H, TT1, TTG, ANR, and naringenin,2-oxoglutarate 3-dioxygenase (an intermediate in flavonoid biosynthesis in plants), all of which are linked to anthocyanin metabolism but also other TFs such as WRKY27 and EGL1-like (Figure 6A). It was reported that *MdWRKY72* promotes anthocyanin accumulation by indirectly binding to the *MdHY5* promoter and directly binding to the *MdMYB1* promoter [33]. Mao et al. [32] reported that the MdHY5-MdWRKY41-MdMYB TF cascade regulates the anthocyanin and proanthocyanidin biosynthesis in red-fleshed apples. Therefore, WRKY27 may interact with MYB12 to regulate anthocyanin accumulation. The bHLH TF EGL1 (enhancer of glabra 1)-like might participate in anthocyanin synthesis as EGL1 was thought to play a role in anthocyanin synthesis in pepper [Zhou 2020], furthermore, EGL3 which was known to regulate anthocyanin biosynthesis in *Arabidopsis* [71]. AHA10, a P-type proton pump which MYB12 interacted with, was reported to play a role in the formation of proanthocyanidins [72], so we speculate that it may indirectly affect the synthesis and transportation of anthocyanins. MYB308-like showed interaction with ANR and naringenin,2-oxoglutarate 3-dioxygenase (Figure 6B), hence, it might affect the final content of anthocyanin. Of other TFs, ALY protein is required for the meiotic cell cycle and is essential for spermatogenesis [73], REVEILLE 3 is an MYB-like TF that plays a photoperiod role in growth regulation [74], and PRP8 and SYF2 are pre-mRNA-splicing-related proteins according to the annotation of the STRING database. All of these proteins can interact with MYB308-like, indicating that MYB308-like might participate in multiple life processes, not only affecting the anthocyanin metabolism. The MYB-related 306-like exhibited interaction with the anthocyanin synthesis structural gene DFR and naringenin,2-oxoglutarate 3-dioxygenase (Figure 6C), suggesting that it might have a function in anthocyanin accumulation. According to the gene annotation by plaBi database, MYB16 encodes a R2R3 MYB protein which is involved in the response to UV-B, and one of its target genes is C4H; this indicated it might participate in anthocyanin accumulation. Moreover, the interaction of the MYB16 protein with TT1 also suggested that they might be involved in anthocyanin biosynthesis. While MYB1R1-like exhibited interaction with MYB16 (Figure 6D,E), suggesting that it might indirectly affect anthocyanin accumulation. Several MYB genes have been reported to regulate anthocyanin biosynthesis by directly or indirectly interacting with the structural genes. For instance, *MdMYB90-like* regulates anthocyanin biosynthesis directly through the activation of anthocyanin biosynthesis genes (*MdCHS* and *MdUFGT*), and indirectly through the activation of other genes (*MdMYB1* and *MdbHLH3*) that activate anthocyanin biosynthesis [23]. The MYB16 and MYB1R1-like proteins’ interaction with other proteins such as KUA1-like, stress-enhanced protein2, GATA7, MYBR2, MYBR7, MYBS3-like, MADS23, HMGB15, HBP-1b(c38)-like, NADH kinase-like, and molybdate transporter 1 indicated that they were associated with diverse functions in plants.

Another TF family, bZIP, was also reported to play a role in anthocyanin biosynthesis in apples. bZIP family TFs *MdHY5* [34] and *MdbZIP44* promoted anthocyanin accumulation by regulating downstream anthocyanin biosynthesis genes in apples [75]. In the current study, we identified a bZIP TF HY5-like (Figure 6F) which was predicted to interact with HY5, a light-induced gene that promotes anthocyanin accumulation by regulating the expression of *MdMYB10* and downstream anthocyanin biosynthesis genes [34]. Therefore, we believe that the HY5-like may also play a vital role in light-induced color change in apple peel. The HY5-like protein showed interaction with the COP1, COP1-like, or isoform proteins, which are known as the RING-finger E3 ubiquitin–protein ligase constitutive photomorphogenesis protein. According to one study in *Arabidopsis*, COP1 is a light-inactivable repressor of photomorphogenic development and negatively regulates HY5 [76]. The COP1 was reported to interact with MdMYB1 to regulate light-induced anthocyanin biosynthesis and red fruit coloration in apples [77]. As is well known, MdMYB1 is a crucial regulator of fruit coloration in apples [11]. Thus, all of the above showed that HY5-like might be a critical TF related to anthocyanin metabolism. Other proteins that interacted with HY5-like were named FHY3-like or FHY3-like isoform. It was reported that FHY3 is a far-red elongated hypocotyl protein involved in regulation of gene expression by the phytochrome A-signaling pathway [78], while the HY5 can also regulate stimulus-induced development of root and hypocotyl [79], so the interaction between HY5-like and FHY3-like (or isoform) proteins suggested that they might be involved in regulating the light signal and the hypocotyl elongation.

**Figure 7:**
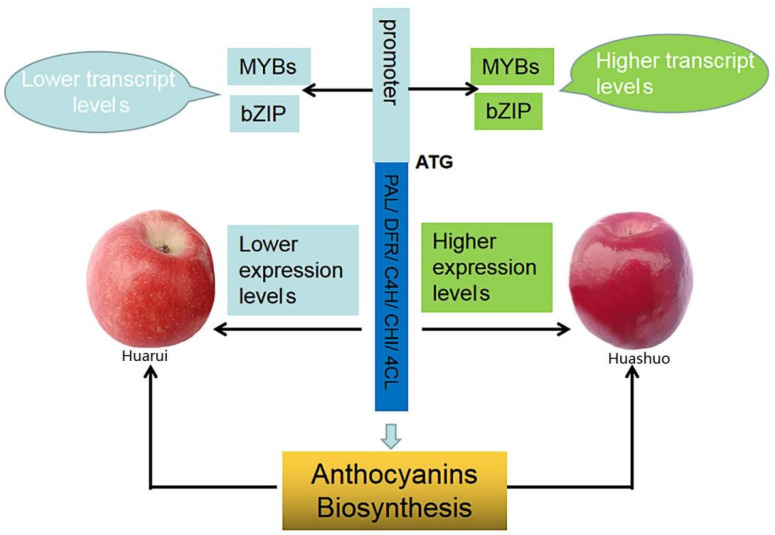
Schematic diagram of differential accumulation of anthocyanin in apple sister lines YS and YR. YS, Huashuo; YR, Huarui.

The role of TFs is essential as they can regulate multiple downstream response genes at the same time. Some of the TFs screened in the current study are known to be associated with the structural gene of anthocyanin synthesis, while others were predicted to interact with other TFs that regulate anthocyanin accumulation. We speculate that the differential expression of the six TFs induce changes in the expression of anthocyanin synthesis-related structural genes and alter endogenous hormones levels, resulting in phenotypic differences between YS and YR (Figure 7); this complicated process warrants further investigation. Most proteins predicted to interact with the DFR, 4CL, and CHI structural genes were anthocyanin-associated structural genes (Figure S3). In addition to the regulation of TFs, protein interactions of structural genes may also influence phenotype; however, further research is required to verify this hypothesis.

## 5. Conclusions

The molecular mechanisms underlying anthocyanin accumulation in YS and YR were analyzed using metabolomics, transcriptomic, qRT-PCR, and interaction analysis. At least 13 anthocyanins were detected in the peels of YS and YR, with cyanidin-3-*O*-galactoside identified as the major anthocyanin. The higher accumulation of anthocyanins in YS, rather than different types of anthocyanins, resulted in a different phenotype compared with YR. The transcriptome data revealed that most anthocyanin biosynthesis-related genes and at least six TFs were significantly correlated with anthocyanin accumulation, which likely contributes to the differential accumulation of anthocyanin in the sister apple lines. Our study findings provide a series of candidate genes with potential applications in the breeding of anthocyanin-rich cultivars.

## Data Availability

Sequence data from this work can be found in the NCBI database (SRA data: PRJNA857470).

## Supplementary Materials

The following supporting information can be downloaded at: https://www.mdpi.com/article/10.3390/life12081246/s1, Figure S1: Number and average expression of genes in each sample of transcriptome; Figure S2: qRT PCR validation of expression levels of 6 randomly selected genes identified by RNA sequencing; Figure S3: Interaction network analysis for MdDFR, Md4CL and MdCHI; Figure S4: Correlation network of metabolites and genes (six key transcription factor) involved in phenylpropanoid and flavonoid biosynthesis in YS and YR; Table S1: Primers used for qRT-PCR; Table S2: Type and content of anthocyanins tested in this project; Table S3: Statistics of all sample quality control data. Table S4: Hormones related to anthocyanin accumulation; Table S5: Main transcription factor information.
