# Supplementary material for: Anthocyanin Accumulation and Molecular Analysis of Correlated Genes by Metabolomics and Transcriptomics in Sister Line Apple Cultivars
Source: Life (Basel). 2022 Aug 16;12(8):1246. doi: 10.3390/life12081246 (PMC9410521; doi:10.3390/life12081246)
Supplement: Supplementary file 1 [file life-12-01246-s001.zip › life-1853202-supplementary.pdf]

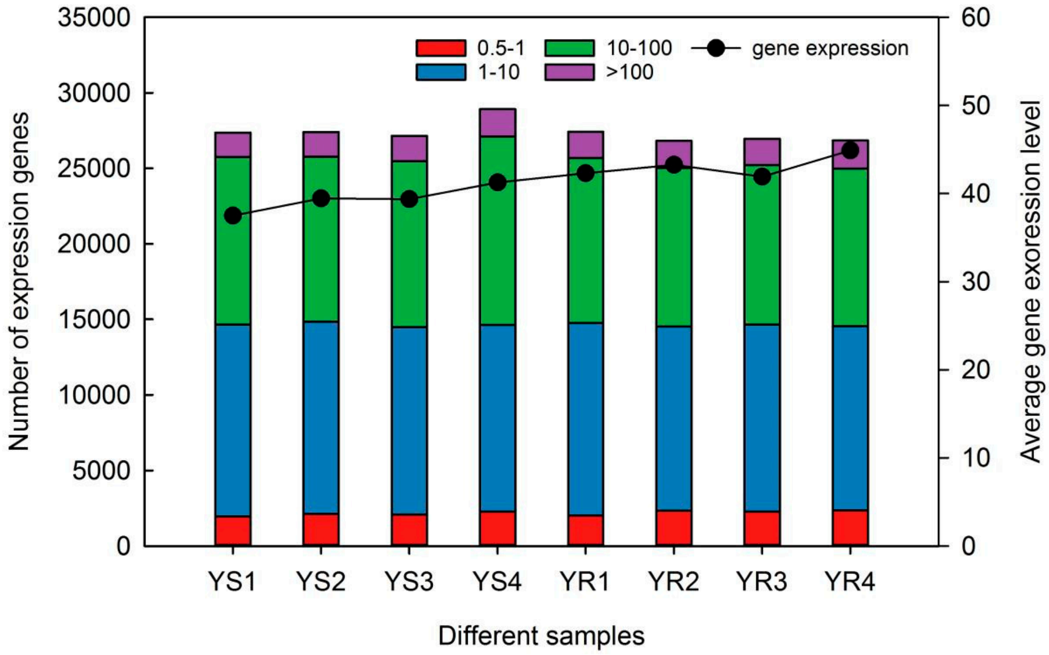

Figure S1 Number and average expression of genes in each sample of transcriptome. YS, Huashuo; YR, Huarui.

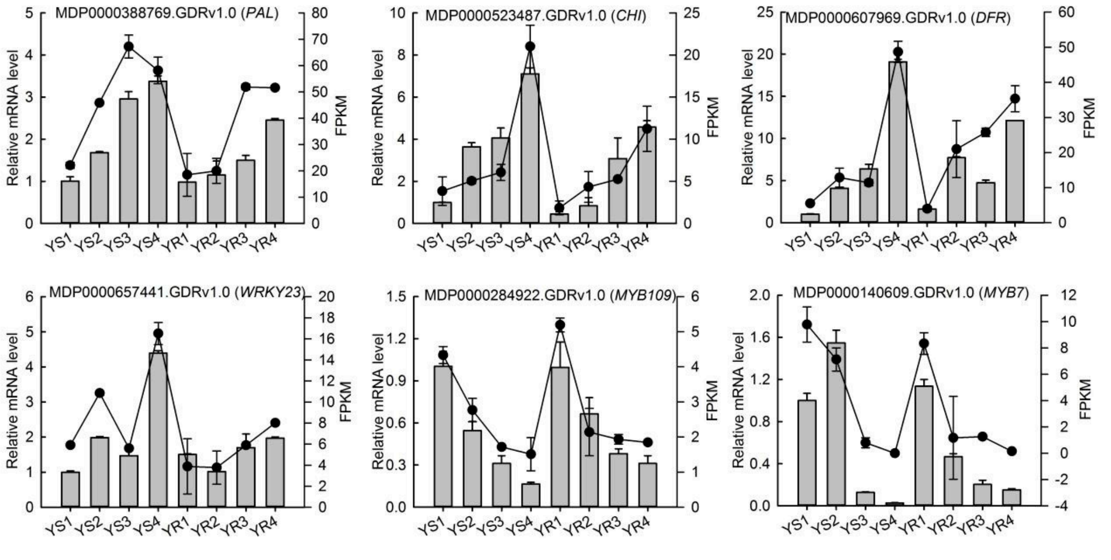

Figure S2 qRT PCR validation of expression levels of 6 randomly selected genes identified by RNA sequencing. The histogram data represent qRT PCR data (left y-axis) and the broken line represents RNA-seq data (right y-axis). The qRT PCR assay had three biological replicates, each with three technical replicates (n = 9). The FPKM value of RNA-seq has three biological repetitions (n = 3). The error bars indicate the standard deviation. The x-axis represents the different samples. YS, Huashuo; YR, Huarui.

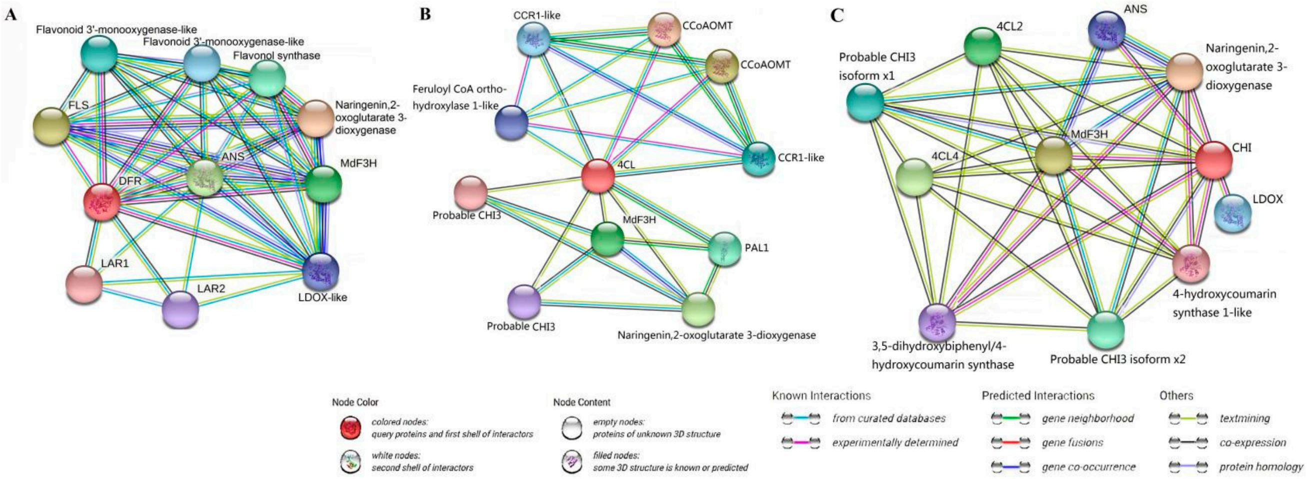

Figure S3 Interaction network analysis for MdDFR, Md4CL and MdCHI. Colored nodes represent different proteins, and the red one was the target protein I typed in. Node content, empty nodes represent proteins of unknown 3D structure, filled nodes represent proteins of 3D structure is known or predicted. Lines of different colors in the middle of nodes represent different relationships of proteins.

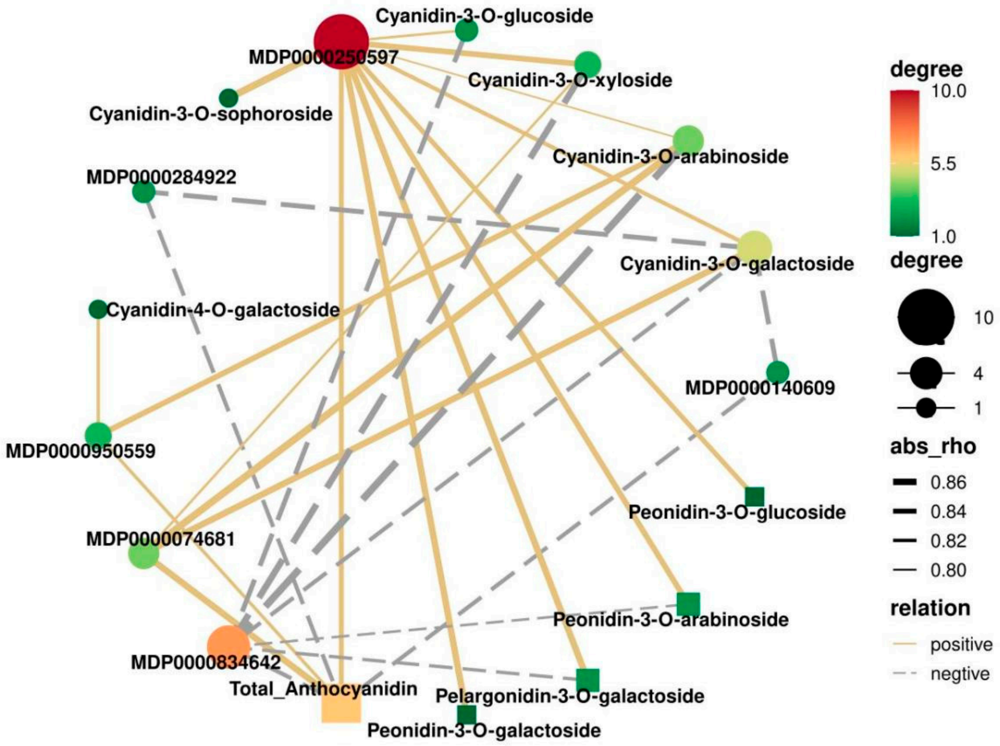

Figure S4 Correlation network of metabolites and genes (six key transcription factor) involved in

phenylpropanoid and flavonoid biosynthesis in YS and YR. Abs_rho represents the Pearson correlation coefficient (r). Degree represents the gene number. Relation represents the correlations with a coefficient (r) value > 0.8 (positive) or < -0.8 (negative). YS, Huashuo; YR, Huarui.

Table S1. Primers used for quantitative real-time PCR (qRT-PCR)

Gene	Forward primer (5'-3')	Reverse primer (5'-3')
Actin	TGACCGAATGAGCAAGGAAATTACT	TACTCAGCTTTGGCAATCCACATC
MDP0000523487 (CHI)	GCTACAAATGCGGTGATAG	CGCCTCCACTACAACCTCC
MDP0000607969 (DFR)	TTGAATCCAAGGACCCCGAG	CTTTTGGTGCTCCTCCACATTC
MDP0000388769 (PAL)	CGAGGAGTGTGACAAGGTGTTCCA	AGGAATGCAGCATGTAAACCGTGAC
MDP0000657441 (WRKY23)	GATGTGGTGAAGAGGGAGGA	ACAGGGAAGTGGGACAACAG
MDP0000284922 (MYB109)	TGATCCAGCAAATGCTTCAG	CCTCAACTCGATCACAGCAA
MDP0000140609 (MYB7)	CATGACCACCAACAACAAGG	CATTAGTCCCCCTGCATTTG

Table S2. Type and content of anthocyanins tested in this project

NO.	Name	Class	RT	Equation	LLOQ	ULOQ	Q1 (Da)	Q3 (Da)	YS1	YS2	YS3	YS4	YR1	YR2	YR3	YR4
1	Cyanidin-3-*O*-galactoside	Cyanidin	6.89	y = 2550.97428 x + 743.84466	5	5000	449.1	287.1	2.41680403	1705.24217	3715.983853	5326.661967	36.70366017	1331.771453	1934.06009	3478.426237
2	Cyanidin-3-*O*-arabinoside	Cyanidin	7.86	y = 1.05803e5 x + 9.08452e4	2	5000	419.1	287.1	0	63.60624863	83.983188	173.5005873	0.363709927	53.84463673	70.88960837	130.8014707
3	Cyanidin-3-*O*-xyloside	Cyanidin	9.64	y = 6.97427e4 x + 6.06251e4	5	5000	419.1	287.1	0.058007778	35.96380077	66.55479137	180.726279	0.864402331	38.53602847	56.38430147	100.1923095
4	Cyanidin-3-*O*-glucoside	Cyanidin	7.43	y = 6.05479e4 x + 7.42416e4	5	5000	449.1	287.1	0	0	13.08211133	51.4382904	0	12.7958415	9.690963853	33.3070245
5	Peonidin-3-*O*-galactoside	Peonidin	8.45	y = 17671.41022 x - 7094.46550	N/A	N/A	463.3	301.1	0	1.472855727	3.139500237	33.9580355	0.222698333	3.850875283	5.447290997	11.48535283
6	Pelargonidin-3-*O*-galactoside	Pelargonidin	7.75	y = 17671.41022 x - 7094.46550	N/A	N/A	433.2	271.1	0	1.34315004	2.198086337	13.1646579	0	2.81587109	2.770928877	6.808400537
7	Cyanidin-3-*O*-sophoroside	Cyanidin	6.87	y = 5.98279e4 x + 18688.60864	1	5000	611.2	287.15	0	0.080658272	0.229901149	1.021511921	0	0.14153142	0.120013313	0.314772673
8	Peonidin-3-*O*-arabinoside	Peonidin	9.37	y = 1.18209e5 x + 8.00277e4	2	5000	433.2	301.1	0	0.008010718	0.035836407	0.539397569	0	0.040858466	0.041091285	0.229422127

9	Pelargonidin-3-O-arabinoside	Pelargonidin	8.7	y = 17671.41022 x - 7094.46550	N/A	N/A	403.1	271.06	0	0	0	0.329387025	0	0.070632929	0.075310046	0.185404744
10	Peonidin-3-*O*-glucoside	Peonidin	8.97	y = 1.75134e5 x + 1.33017e5	2	5000	463.3	301.1	0	0	0.019182982	0.147718811	0	0.005988209	0.006581485	0.023278365
11	Cyanidin-3-*O*-rutinoside	Cyanidin	8.08	y = 17671.41022 x - 7094.46550	N/A	N/A	595.17	287.1	0	0.12337751	0.090298809	0.368671285	0	0.49530838	0.125129403	0.221357853
12	Delphinidin-3-*O*-glucoside	Delphinidin	6.43	y = 6.68665e4 x + 13056.59708	5	5000	465.1	303.1	0	0	0	0	0	0	0	0.311386889
13	Delphinidin-3-*O*-arabinoside	Delphinidin	6.99	y = 17671.41022 x - 7094.46550	N/A	N/A	435.5	303.1	0.049599748	0.03103483	0.028638458	0	0.045880427	0	0.032207366	0

Note: LLOQ and ULOQ represent the lowest concentration and the highest concentration of the corresponding substance detected in the sample respectively. The substance with LLOQ and ULOQ value were quantified through the retention time (RT) and Q1, Q3 value of standard sample, and substances without values were semi quantitative. Semi quantitative analysis was mainly based on the fragmentation mode of mass spectrometry and the peak rule of liquid phase of substances with standards. Q1, Relative molecular mass of original ion. Q3, Relative molecular mass of the characteristic fragment after cleavage. The unit of anthocyanin content was μg/g. YS, Huashuo; YR, Huarui.

Table S3. Statistics of all sample quality control data

Sample NO.	Total Reads Count	Total Bases Count (bp)	Average Read Length (bp)	Q20 Bases Ratio (%)	Q30 Bases Ratio (%)	GC Bases Ratio (%)
1	50939656.67	7145200980	140.32	97.48%	90.48%	47.11%
2	58922644.00	8273328673	140.41	97.94%	92.51%	46.91%
3	50589868.67	7127723224	141.1333333	97.88%	91.64%	47.29%
4	62619902.67	8786949099	140.3033333	98.28%	93.58%	46.86%
5	48690526.67	6875857480	141.2066667	97.33%	90.00%	47.39%
6	57012886.00	8029186252	140.81	98.17%	93.22%	47.02%
7	55532436.67	7754430281	139.5833333	97.94%	92.38%	47.09%
8	61891340.67	8466435258	137.0066667	98.31%	93.76%	47.68%

Note: 1-8 sequential represent YS1-4, YR1-4. YS, Huashuo; YR, Huarui.

Table S4. Hormones related to anthocyanin accumulation

Category	ID	Annotation	Accession	Anthocyanin species (>0.8)	Correlation coefficient
ABA	MDP0000058337	HYR1, a UDP glycosyltransferase	at3g21760	1,2,3,4,5,6,8,9,total	0.82/0.94/0.86/0.93/0.8/0.88/0.85/0.91/0.69/0.83
	MDP0000242552	UDP-glucosyl transferase 71C4	at1g07250	1,2,3,4,5,6,7,8,9,10,total	0.86/0.89/0.91/0.93/0.91/0.92/0.92/0.93/0.88/0.88/0.87
	MDP0000715898	HYR1, a UDP glycosyltransferase	at3g21760	1,2,3,4,5,6,8,9,total	0.82/0.94/0.86/0.93/0.82/0.89/0.87/0.92/0.72/0.83
	MDP0000744887	NCED5(9-cis-epoxycarotenoid dioxygenase5）	at4g19170	1,2,3,4,5,6,7,8,10,total	0.93/0.88/0.93/0.89/0.83/0.86/0.89/0.82/0.82/0.93
	MDP0000231674	Protein phosphatase 2C family protein	at5g57050	2,3,4,5,6,8,9,total	0.9/0.82/0.89/0.81/0.87/0.83/0.92/0.8
	MDP0000162721	GRAM domain family protein	at5g13200	1,2,3,4,5,6,7,8,9,10,total	0.86/0.91/0.93/0.94/0.95/0.96/0.95/0.97/0.92/0.92/0.87
	MDP0000169913	GRAM domain family protein	at5g13200	1,2,3,4,5,6,7,8,9,10,total	0.87/0.86/0.88/0.89/0.91/0.91/0.89/0.87/0.9/0.86/0.87
	MDP0000250947	ABA-responsive element binding protein 3	at2g41070	1,2,3,4,6,8,9,total	-0.87/-0.89/-0.87/-0.9/-0.85/-0.8/-0.83/-0.88
	MDP0000463117	GRAM domain family protein	at5g13200	1,2,3,4,5,6,7,8,9,10,total	0.9/0.83/0.9/0.85/0.82/0.84/0.88/0.81/0.83/0.9
	MDP0000821241	GRAM domain family protein	at5g23370	1,2,3,4,5,6,7,8,total	0.88/0.86/0.89/0.86/0.8/0.84/0.84/0.82/0.88
auxin	MDP0000160094	a methyl IAA esterase	at3g10870	1,2,4,total	-0.85/-0.83/-0.81/-0.85
	MDP0000281064	IAA-leucine resistant (ILR)-like 2	at3g02875	1,2,3,4,5,6,7,8,9,10,total	0.9/0.97/0.95/0.98/0.95/0.98/0.93/0.97/0.97/0.89/0.91
	MDP0000319659	IAA-leucine resistant (ILR)-like gene 5	at1g51760	1,2,3,4,5,6,7,8,9,10,total	0.88/0.88/0.92/0.88/0.9/0.91/0.92/0.91/0.85/0.88/0.88

	MDP0000026750	ARG1-like 1	at1g68370	1,2,total	0.81/0.81/0.81
	MDP0000125975	GRR1-like protein 1	at3g62980	1,2,total	-0.83/-0.82/-0.83
	MDP0000175425	like AUXIN RESISTANT 1	q9fel7|lax2_medtr	1,2,total	-0.8/-0.8/-0.8
	MDP0000234528	Auxin efflux carrier family protein	at1g70940	1,2,3,4,total	0.84/0.87/0.82/0.85/0.84
	MDP0000385305	sorting nexin 2A	at5g58440	1,2,3,4,6,total	0.9/0.87/0.87/0.83/0.8/0.9
	MDP0000129063	O-fucosyltransferase family protein	at5g65470	1,2,4,total	-0.81/-0.83/-0.83/-0.81
	MDP0000148780	SAUR-like auxin-responsive protein family	p33083|ax6b_soybn	1,2,3,total	-0.86/-0.82/-0.82/-0.85
	MDP0000148873	Aluminium induced protein with YGL and LRDR motifs	at4g27450	1,2,3,4,5,6,7,8,9,10,total	0.83/0.92/0.89/0.96/0.94/0.96/0.89/0.95/0.98/0.86/0.84
	MDP0000231245	Auxin-responsive GH3 family protein	q60ey1|gh36_orysa	1,2,3,4,5,6,8,9,total	0.85/0.95/0.87/0.93/0.8/0.88/0.84/0.89/0.86
	MDP0000234699	O-fucosyltransferase family protein	at5g65470	1,2,4,9,total	-0.8/-0.85/-0.86/-0.81/-0.81
	MDP0000264686	SAUR-like auxin-responsive protein family	at4g12410	1,2,3,4,5,6,7,8,9,total	0.88/0.92/0.88/0.91/0.87/0.92/0.83/0.87/0.92/0.89
	MDP0000282950	O-fucosyltransferase family protein	at3g30300	1,2,3,total	-0.86/-0.85/-0.82/-0.86
	MDP0000307308	O-fucosyltransferase family protein	at1g22460	1,2,3,total	-0.86/-0.86/-0.8/-0.86
	MDP0000612130	ATB2	at1g60710	1,2,3,4,5,6,7,8,9,total	0.92/0.94/0.92/0.93/0.82/0.89/0.83/0.85/0.84/0.92
	MDP0000811081	Auxin-responsive GH3 family protein	at4g03400	1,2,3,4,6,9,total	-0.84/-0.9/-0.83/-0.89/-0.82/-0.84/-0.85
	MDP0000813577	Aluminium induced protein with YGL and LRDR motifs	gnl|cdd|48483	2,3,4,5,6,8,9,total	0.92/0.84/0.92/0.81/0.88/0.87/0.9/0.8
	MDP0000876321	an auxin response factor	at1g19220	1,2,total	-0.86/-0.83/-0.85
BR	MDP0000498540	Cytochrome P450 superfamily protein	at3g50660	1,2,3,6,9,total	-0.84/-0.81/-0.8/-0.83/-0.81/-0.84
	MDP0000307964	brassinosteroid-6-oxidase 1	q69f95|c85a_phavu	1,2,3,4,total	-0.89/-0.85/-0.83/-0.82/-0.89
	MDP0000307964	brassinosteroid-6-oxidase 1	q69f95|c85a_phavu	1,2,3,4,total	-0.89/-0.85/-0.83/-0.82/-0.89
	MDP0000465581	Fatty acid hydroxylase superfamily protein	q9zt29|sc5d_tobac	1,2,3,4,5,6,7,8,9,total	-0.84/-0.87/-0.85/-0.89/-0.81/-0.85/-0.81/-0.81/-0.83/-0.84
	MDP0000191389	Squalene monooxygenase	o48651|erg1_pangi	1,2,total	0.82/0.83/0.82
	MDP0000278941	cyclopropyl isomerase (CPI1)	at5g50375	1,2,3,4,6,total	-0.88/-0.87/-0.85/-0.85/-0.8/-0.88
	MDP0000297861	Squalene monooxygenase	o48651|erg1_pangi	1,2,3,4,6,9,total	-0.91/-0.92/-0.87/-0.89/-0.85/-0.83/-0.91
	MDP0000413935	Squalene monooxygenase	o48651|erg1_pangi	1,2,3,4,5,6,7,8,9,total	-0.9/-0.87/-0.89/-0.88/-0.84/-0.87/-0.85/-0.84/-0.81/-0.9
	MDP0000857446	Squalene monooxygenase	o48651|erg1_pangi	1,2,3,4,5,6,7,8,9,10,total	0.84/0.9/0.89/0.92/0.93/0.95/0.89/0.95/0.95/0.86/0.85

	MDP0000157003	BRI1 like	at4g39400	1,total	-0.81/-0.8
	MDP0000295859	GSK3-like protein kinase	at2g30980	1,2,3,4,6,7,8,total	-0.8/-0.85/-0.85/-0.83/-0.82/-0.81/-0.81/-0.81
	MDP0000130173	BES1/BZR1 homolog 4 (BEH4)	at1g78700	1,2,3,4,total	0.86/0.85/0.8/0.81/0.86
CTK	MDP0000166723	cytokinin oxidase 4	gnl|cdd|72681	1,2,3,4,5,6,7,8,9,10,total	0.9/0.96/0.95/0.97/0.93/0.97/0.92/0.95/0.93 /0.86/0.91
	MDP0000299496	UDP-glucosyl transferase 85A2 (UGT85A2)	at1g22360	1,2,3,4,6,9,total	-0.8/-0.89/-0.81/-0.84/-0.82/-0.86/-0.81
	MDP0000312902	cytokinin oxidase 2	at4g29740	1,2,3,4,5,6,7,8,9,10,total	0.83/0.92/0.91/0.95/0.94/0.96/0.92/0.98/0.95/0.89 /0.84
	MDP0000813186	UDP-glucosyl transferase 85A7 (UGT85A7)	at1g22340	1,2,3,4,5,6,7,8,9,10,total	0.82/0.88/0.91/0.92/0.98/0.96/0.97/0.98/0.94/0.97/0.83
ETH	MDP0000148169	ETO1-like 2	at3g51770	1,2,3,4,6,7,total	-0.9/-0.9/-0.9/-0.84 /-0.84 /-0.8/-0.91
	MDP0000188827	similar to 2-oxoglutarate-dependent dioxygenase	at1g06650	1,2,4,9,total	0.82/0.88/0.83/0.81 /0.82
	MDP0000205515	2-oxoglutarate-dependent dioxygenase	at1g06650	1,2,4,9,total	0.8/0.88/0.83/0.82/0.8
	MDP0000265117	Pyridoxal-5'-phosphate-dependent enzyme family protein	at1g48420	1,2,3,4,6,9,total	0.92/0.92/0.88/0.9/0.87/0.86/0.92
	MDP0000281562	similar to 2-oxoglutarate-dependent dioxygenase	at1g06650	1,2,4,total	0.8/0.85/0.8/0.8
	MDP0000644549	2-oxoglutarate (2OG) and Fe(II)-dependent oxygenase superfamily protein	at1g49390	1,2,3,4,6,total	-0.9/-0.9/-0.85/-0.86/-0.8/0.9
	MDP0000535274	ACC synthase 10	at5g51690	1,2,3,4,6,9,total	0.8/0.9/0.8/0.89/0.81/0.84/0.81
	MDP0000127054	Integrase-type DNA-binding superfamily protein	at5g25190	2,3,4,5,6,7,8,9,total	0.91/0.85/0.91/0.84/0.9/0.8/0.88/0.920.8
	MDP0000161249	Integrase-type DNA-binding superfamily protein	at5g07580	1,2,3,total	-0.89/-0.84/-0.85/-0.89
	MDP0000178263	Integrase-type DNA-binding superfamily protein	at5g11190	1,2,3,4,total	-0.81/-0.85/-0.8/-0.87/-0.82
	MDP0000226115	ethylene responsive element binding factor 2	q40479|erf2_tobac	1,2,3,4,5,6,7,8,9,total	0.85/0.96/0.9/0.96/0.89/0.94/0.85/0.93/0.96/0.86
	MDP0000257135	Signal transduction histidine kinase	at2g40940	1,2,3,4,6,9,,total	0.88/0.93/0.86/0.9/0.87/0.89/0.89
	MDP0000287350	Integrase-type DNA-binding superfamily protein	at5g25390	1,2,3,4,5,6,8,9,total	-0.87/-0.93/-0.88/-0.92/-0.8/-0.88/-0.83/-0.87/-0.87
	MDP0000313063	Plant protein of unknown function	gnl|cdd|66792	1,2,3,4,5,6,7,8,9,10,total	0.8/0.86/0.89/0.9/0.97/0.95/0.95/0.98/0.93/0.95/0.81
	MDP0000629527	ethylene responsive element binding factor 3	gnl|cdd|47695	1,2,3,4,6,9,total	0.86/0.91/0.84/0.9/0.84/0.85/0.87

	MDP0000689946	ethylene responsive element binding factor 1	at5g47220	1,2,3,4,5,6,7,8,9,total	0.87/0.95/0.91/0.95/0.87/0.93/0.85/0.91/0.93/0.87
	MDP0000769656	Plant protein of unknown function	gnl|cdd|66792	1,2,3,4,5,6,7,8,9,10,total	0.81/0.84/0.9/0.88/0.97/0.94/0.97/0.97/0.9/0.98/0.82
	MDP0000839828	ethylene response factor 7	gnl|cdd|47695	1,2,3,4,5,6,7,8,9,10,total	0.98/0.92/0.95/0.89/0.86/0.91/0.89 0.85/0.84/0.82/0.97
	MDP0000842644	Integrase-type DNA-binding superfamily protein	at5g07580	1,total	-0.83/-0.82
GA	MDP0000716315	GA3 oxidase	at1g15550	1,2,3,4,6,9,total	0.81/0.9/0.84/0.89/0.82/0.8/0.82
	MDP0000137705	GA2 oxidase	q9sq80|g2ox1_pea	1,2,3,4,5,6,7,8,9,10,total	0.93/0.91/0.96/0.93/0.95/0.95/0.97 0.94/0.89/0.94/0.93
	MDP0000185333	GA2 oxidase	q9sq80|g2ox1_pea	1,2,3,4,5,6,7,8,9,10,total	0.9/0.86/0.93/0.9/0.93/0.92/0.96/0.91/0.84/0.94/0.9
	MDP0000129445	ARM repeat superfamily protein	at5g37490	1,2,3,4,5,6,7,8,9,10,total	0.92/0.93/0.97/0.95/0.94/0.96/0.96/0.95/0.9/0.92/0.93
	MDP0000242829	RGA-like 2	gnl|cdd|67150	1,2,3,4,6,9,total	-0.9/-0.9/-0.86/-0.88/-0.83/-0.81/-0.9
	MDP0000319522	alpha/beta-Hydrolases superfamily protein	at3g63010	1,2,3,4,5,6,7,8,9,10,total	0.81/0.88/0.86/0.92/0.88/0.9/0.85/0.91/0.89 /0.81/0.82
	MDP0000669451	GRAS family transcription factor family protein	q84tq7|gai_goshi	1,2,4,9,total	-0.83/-0.84/-0.8/-0.8/-0.83
	MDP0000950387	DELLA protein GAI	q84tq7|gai_goshi	1,2,4,9,total	-0.82/-0.84/-0.82/-0.81/-0.82
	MDP0000669451	DELLA protein GAI	q84tq7|gai_goshi	1,2,4,9,total	-0.83/-0.84/-0.8/-0.8/-0.83
JA	MDP0000125303	lipoxygenase 1	gnl|cdd|84680	1,2,3,4,5,6,7,8,9,10,total	0.83/0.93/0.91/0.95/0.94/0.96/0.92/0.97/0.95/0.88/0.84
	MDP0000204470	LOX5	at3g22400	1,2,3,4,5,6,7,8,9,10,total	-0.89/-0.94/-0.92/-0.97/-0.91/-0.95/-0.9/-0.92/-0.94/-0.85/-0.89
	MDP0000215405	PLAT/LH2 domain-containing lipoxygenase family protein	at1g67560	1,2,3,4,5,6,7,8,9,10,total	-0.88/-0.87/-0.9/-0.89/-0.89/-0.9/-0.89/-0.88/-0.86/-0.85/-0.88
	MDP0000264666	Lipoxygenase 1	p37831|lox1_soltu	1,2,3,4,5,6,7,8,9,10,total	0.82/0.87/0.9/0.89/0.96/0.95/0.94/0.97/0.93/0.93/0.82
	MDP0000423544	Lipoxygenase 1	p37831|lox1_soltu	1,2,3,4,5,6,7,8,9,10,total	0.82/0.89/0.88/0.9/0.93/0.94/0.89/0.95/0.94/0.86/0.82
	MDP0000450991	Lipoxygenase 1	p37831|lox1_soltu	1,2,3,4,6,8,9,total	0.85/0.95/0.86/0.92/0.88/0.83/0.9/0.85
	MDP0000520035	Lipoxygenase 1	p37831|lox1_soltu	1,2,3,4,5,6,7,8,9,10,total	0.83/0.89/0.92/0.93/0.98/0.96/0.97/0.99/0.94/0.96/0.84
	MDP0000548790	lipoxygenase	at1g55020	1,2,3,4,6,9,total	0.83/0.91/0.81/0.86/0.8/0.82/0.84
	MDP0000923670	Lipoxygenase 1	p37831|lox1_soltu	1,2,3,4,5,6,7,8,9,10,total	0.81/0.89/0.86/0.91/0.89/0.92/0.85/0.91/0.92/0.81/0.82
	MDP0000273180	12-oxophytodienoate reductase 2	at2g06050	1,2,3,4,5,6,7,8,9,10,total	0.8/0.85/0.86/0.85/0.82/0.85/0.85/0.84/0.81/0.81/0.81
	MDP0000645422	12-oxophytodienoate reductase 2	at1g76680	1,7,total	0.81/0.8/0.81
SA	MDP0000250047	UGT74E2	at1g05680	1,2,3,4,5,6,7,8,9,10,total	0.81/0.93/0.89/0.95/0.91/0.94/0.87/0.95/0.95/0.83/0.83
	MDP0000305536	no description available	gnl|cdd|86349	1,2,3,4,5,6,7,8,9,10,total	0.9/0.95/0.94/0.96/0.91/0.94/0.9/0.93/0.91/0.85/0.91

	MDP0000312765	no description available	gnl|cdd|86349	1,2,3,4,5,6,7,8,9,10,total	0.87/0.94/0.91/0.95/0.94/0.96/0.89/0.94/0.98/0.84/0.88
	MDP0000250047	UGT74E2	at1g05680	1,2,3,4,5,6,7,8,9,10,total	0.81/0.93/0.89/0.95/0.91/0.94/0.87/0.95/0.95/0.83/0.83

Note: Anthocyanin category No. refer to table 2

Table S5. Main transcription factor information

Category	Gene ID	Anthocyanin species (>0.8)	Correlation coefficient
AP2/EREBP	MDP0000155743	2,3,4,5,6,7,8,9,10,total	0.84/0.9/0.89/0.98/0.94/0.98/0.97/0.91/0.99/0.8
	MDP0000178263	1,2,3,4,total	-0.81/-0.85/-0.8/-0.87/0.82
	MDP0000218344	1,2,3,4,5,6,7,8,9,total	0.87/0.96/0.9/0.93/0.82/0.9/0.81/0.87/0.89/0.88
	MDP0000242979	1,2,3,4,5,6,7,8,9,10,total	0.85/0.91/0.92/0.95/0.96/0.96/0.94/0.97/0.95/0.92/0.86
	MDP0000287350	1,2,3,4,5,6,7,8,9,total	-0.87/-0.93/-0.88/-0.92/-0.8 /-0.88/-0.79/-0.83/-0.87/-0.87
	MDP0000316694	1,2,3,4,5,6,7,8,9,10,total	0.9/0.95/0.95/0.96/0.91/0.95/0.91/0.94/0.91/0.86/0.91
	MDP0000759299	1,2,3,4, 6, 8,9,total	-0.84/-0.92/-0.85/-0.89/-0.86/-0.81/-0.88/-0.85
	MDP0000854039	1,total	-0.82/-0.82
ARF	MDP0000173151	1,2,3,4,5,6,7,8,9,10,total	-0.95/-0.9/-0.95/-0.87/-0.85/-0.89/-0.89/-0.85/-0.81/-0.82/-0.95
	MDP0000179650	1,2,4,6,9,total	-0.82/-0.87/-0.84/-0.8/-0.83/-0.83
	MDP0000211459	1,2,3,4,6,8,9,total	0.83/0.94/0.85/0.93/0.88/0.84/0.9/0.84
	MDP0000310875	1,2,3,6,9,total	-0.84/-0.86/-0.81/-0.81/-0.82/-0.84
	MDP0000412781	1,2,3,4,6,9,total	-0.81/-0.85/-0.8/-0.8//-0.84/-0.86/-0.82
	MDP0000876321	1,2,total	-0.86/-0.83/-0.85
ARR	MDP0000224740	1,2,total	-0.82/-0.8/-0.82
	MDP0000290818	1,2,total	-0.81/-0.83/-0.81
	MDP0000585643	1,2,3,4,6,9,total	-0.8/-0.89/-0.8/-0.86 /-0.84/-0.88/-0.81
bHLH	MDP0000154272	1,2,total	-0.82/-0.84/-0.82
	MDP0000214472	1,2,3,4,6,7,8,total	0.8/0.81/0.83/0.86/0.81/0.82/0.81/0.8
	MDP0000231650	1,2,3,4,6,9,total	-0.88/-0.88/-0.84/-0.82/-0.83/-0.83/-0.88
	MDP0000261874	1,2,3,total	-0.9/-0.84/-0.81/-0.89
	MDP0000312274	1,2,3,4,5,6,7,8,9,total	0.85/0.95/0.89/0.95/0.88/0.93/0.83/0.9/0.97/0.86
	MDP0000320691	1,total	-0.84/-0.83
	MDP0000439540	1,2,3,4,6,9,total	-0.86/-0.9/-0.86/-0.86/-0.84 /-0.82/-0.87
	MDP0000779358	1,3,total	-0.86/-0.81/-0.86
	MDP0000799392	1,2,3,4,total	-0.83/-0.84/-0.8/-0.83/-0.83
	MDP0000829998	1,2,3,4,6,total	-0.81/-0.86/-0.83/-0.87/-0.8/-0.82
C2C2	MDP0000151848	1,total	-0.86/-0.86
	MDP0000194889	1,total	-0.8/-0.8
	MDP0000321180	1,2,9,total	-0.83/-0.82/-0.8/-0.83
	MDP0000551876	1,2,3,4,6,7,total	0.91/0.88/0.88/0.87/0.83/0.8/0.91

	MDP0000565292	1,2,3,4,6,9,total	-0.9/-0.9/-0.87/-0.88 /-0.86/-0.84/-0.91
	MDP0000697407	1,3,5,6,7,9,total	0.81/0.8/0.82/0.82/0.8/0.81/0.81
	MDP0000713113	1,2,3,4,6,9,total	-0.89/-0.92/-0.86/-0.85/-0.85/-0.84/-0.89
	MDP0000901915	1,2,4,9,total	-0.8/-0.84/-0.8/-0.82/-0.8
	MDP0000201120	1,2,3,6,7,total	-0.87/-0.81/-0.87/-0.81/-0.84/-0.87
	MDP0000547254	1,total	-0.84/-0.84
C2H2	MDP0000122321	1,2,total	-0.85/-0.82/-0.85
	MDP0000128287	1,2,3,4,total	0.84/0.85/0.83/0.82/0.84
	MDP0000137826	1,2,3,4,5,6,7,8,9,10,total	0.8/0.85/0.9/0.9/0.99/0.95/0.97/0.98/0.93/0.98/0.81
	MDP0000190415	1,3,4,7,total	0.83/0.85/0.82/0.82/0.84
	MDP0000198015	1,3,4,7,total	0.83/0.82/0.8/0.81/0.83
	MDP0000200608	1,2,3,4,6,7,total	0.88/0.82/0.87/0.81/0.81/0.83/0.88
	MDP0000319225	1,3,total	0.83/0.8/0.82
	MDP0000453148	2,3,4,5,6,7,8,9,total	0.88/0.85/0.91/0.85/0.88/0.82/0.89/0.87/0.8
	MDP0000535412	1,2,3,4,6,9,total	-0.87/-0.91/-0.84/-0.87/-0.84/-0.85/-0.87
	MDP0000551046	1,2,total	-0.85/-0.8/-0.85
	MDP0000573176	1,2,3,4,5,6,7,8,total	0.87/0.87/0.89/0.88/0.8/0.84/0.84/0.82/0.88
	MDP0000610922	1,2,3,4,5,6,7,8,9,total	0.87/0.94/0.89/0.92/0.84/0.91/0.82/0.88/0.91/0.88
	MDP0000762756	1,2,3,4,5,6,7,8,9,10,total	0.9/0.91/0.91/0.93/0.89/0.92/0.88/0.89/0.89/0.82/0.91
	MDP0000807856	1,total	-0.82/-0.81
	MDP0000853499	1,2,3,4,5,6,7,8,9,10,total	0.84/0.91/0.9/0.92/0.93/0.94/0.9/0.93/0.95/0.86/0.85
C3H	MDP0000171955	1,2,3,total	-0.87/-0.8/-0.81/-0.87
	MDP0000673185	1,2,3,4,5,6,7,8,9,10,total	-0.8/-0.84/-0.85/-0.82/-0.86 /-0.87/-0.86/-0.87/-0.84/-0.83/-0.8
E2F/DP	MDP0000232265	1,2,3,4,total	-0.85/-0.84/-0.8/-0.81/-0.85
	MDP0000316055	1,2,3,4,6,9,total	0.91/0.9/0.87/0.85/0.86/0.84/0.91
G2-like	MDP0000124687	1,2,total	-0.85/-0.83/-0.85
	MDP0000182154	1,2,3,total	-0.89/-0.8/-0.81/-0.88
	MDP0000202657	1,total	0.86/0.86
	MDP0000248927	1,2,3,4,6,9,total	0.8/0.88/0.8/0.85/0.8/0.81/0.8
	MDP0000320628	1,total	-0.8/-0.8
	MDP0000526864	1,2,3,total	-0.89/-0.83/-0.82/-0.89
GRAS	MDP0000669451	1,2,4,total	-0.83/-0.84/-0.8/-0.83
	MDP0000796348	1,2,total	-0.85/-0.87/-0.85
	MDP0000827722	1,2,3,total	-0.84/-0.81/-0.8/-0.83
	MDP0000950387	1,2,4,total	-0.82/-0.84/-0.82/-0.82
HB	MDP0000124827	1,2,3,4,5,6,7,8,9,10,total	-0.9/-0.89/-0.92/-0.91/-0.91 /-0.93/-0.91/-0.91/-0.89/-0.87/-0.9
	MDP0000222305	2,4,total	0.87/0.83/0.8

	MDP0000272542	1,2,total	-0.85/-0.83/-0.85
	MDP0000316497	1,2,3,4,5,6,7,8,9,10,total	0.88/0.92/0.93/0.93/0.96/0.96/0.93/0.95/0.96/0.91/0.88
	MDP0000405958	2,3,4,6,8,9,total	0.88/0.8/0.85/0.86/0.81/0.9/0.8
	MDP0000423596	1,2,3,total	-0.9/-0.87/-0.83/-0.9
	MDP0000596661	1,2,total	-0.85/-0.82/-0.85
	MDP0000840574	1,2,total	-0.81/-0.81/-0.81
	MDP0000899816	1,2,3,4,5,6,7,8,9,total	0.86/0.96/0.89/0.95/0.85/0.92/0.81/0.88/0.95/0.87
HSF	MDP0000155579	1,2,3,4,6,7,total	0.85/0.8/0.85/0.82/0.8/0.83/0.85
	MDP0000202716	1,2,3,4,5,6,7,8,9,10,total	0.87/0.94/0.94/0.96/0.97/0.98/0.96/0.99/0.96/0.93/0.88
	MDP0000319456	1,2,3,4,6,9,total	0.8/0.87/0.81/0.86/0.85/0.88/0.81
	MDP0000319910	1,2,3,4,5,6,7,8,9,total	0.91/0.93/0.91/0.9/0.87/0.92/0.85/0.86/0.92/0.91
MYB	MDP0000140609	1,total	-0.84/-0.83
	MDP0000164048	1,2,3,4,total	-0.89/-0.87/-0.88/-0.82/-0.89
	MDP0000194307	1,2,3,4,6,8,9,total	-0.91/-0.93/-0.88/-0.91/-0.88/-0.8/-0.87/-0.91
	MDP0000209974	1,2,3,4,6,8,total	0.82/0.87/0.86/0.84/0.83/0.82/0.83
	MDP0000250597	1,2,3,4,5,6,7,8,10,total	0.83/0.8/0.85/0.81/0.86/0.86/0.87/0.85/0.84/0.84
	MDP0000282792	1,2,3,4,6,9,total	-0.86/-0.87/-0.8/-0.82/-0.81 /-0.84/-0.86
	MDP0000284922	1,total	-0.84/-0.83
	MDP0000478512	1,total	-0.83/-0.82
	MDP0000543585	1,total	-0.84/-0.83
	MDP0000659260	1,2,3,4,6,8,9,total	0.87/0.94/0.86/0.9/0.85/0.8/0.84/0.87
	MDP0000742771	1,2,3,4,6,9,total	-0.85/-0.9/-0.83/-0.88/-0.83/-0.85/-0.86
	MDP0000934638	1,2,3,4,5,6,7,8,9,10,total	0.93/0.91/0.93/0.92/0.87/0.91/0.89/0.85/0.87/0.83/0.93
	MDP0000950559	1,2,total	0.82/0.85/0.82
MYB-related	MDP0000074681	1,2,3,total	0.86/0.87/0.81/0.86
	MDP0000208448	1,2,3,4,6,total	0.81/0.83/0.81/0.8/0.8/0.82
	MDP0000209471	1,3,total	-0.89/-0.81-0.89
	MDP0000241185	1,2,3,4,6,8,9,total	-0.88/-0.93/-0.87/-0.87/-0.86/-0.8/-0.86/-0.88
	MDP0000255970	1,2,3,4,5,6,7,8,9,total	0.88/0.96/0.89/0.93/0.82/0.9/0.8/0.86/0.89/0.89
	MDP0000273563	1,2,3,4,total	-0.81/-0.85/-0.82/-0.84/-0.81
NAC	MDP0000158717	1,2,3,4,total	-0.88/-0.85/-0.81/-0.81/-0.87
	MDP0000228792	1,2,3,total	-0.84/-0.82/-0.81/-0.84
	MDP0000309382	1,2,3,4,5,6,7,8,9,10,total	0.93/0.97/0.95/0.96/0.92/0.97/0.89/0.93/0.95/0.82/0.94
	MDP0000404409	1,2,3,4,6,9,total	0.87/0.94/0.87/0.9/0.84/0.84/0.87
TCP	MDP0000523096	1,2,total	-0.83/-0.8/-0.82
	MDP0000763497	1,2,3,4,6,9,total	-0.86/-0.85/-0.84/-0.82/-0.85/-0.83/-0.86
	MDP0000920127	1,total	-0.83/-0.82

Triple-Helix	MDP0000156139	1,2,3,4,6,9,total	-0.83/-0.86/-0.8/-0.83/-0.81/-0.83/-0.83
	MDP0000158598	1,2,total	-0.84/-0.84/-0.84
	MDP0000209313	1,2,3,4,5,6,7,8,9,total	-0.93/-0.93/-0.93/-0.91/-0.85/-0.9/-0.86/-0.85/-0.87/-0.93
	MDP0000271048	1,2,3,4,6,total	-0.95/-0.88/-0.89/-0.82/-0.83/-0.95
WRKY	MDP0000119590	1,2,3,4,5,6,7,8,9,10,total	0.84/0.87/0.91/0.88/0.91/0.91/0.93/0.94/0.86/0.9/0.84
	MDP0000129882	1,2,3,4,6,total	0.87/0.82/0.84/0.83/0.82/0.86
	MDP0000144730	1,2,3,4,6,8,9,total	0.83/0.91/0.86/0.91/0.86/0.84
	MDP0000146390	1,2,3,4,5,6,7,8,9,10,total	0.9/0.91/0.92/0.88/0.89/0.93/0.88/0.89/0.9/0.82/0.91
	MDP0000177906	1,2,3,4,5,6,7,8,9,total	0.92/0.97/0.93/0.96/0.89/0.95/0.86/0.91/0.94/0.93
	MDP0000231668	1,2,3,4,5,6,7,8,9,10,total	0.85/0.95/0.92/0.97/0.93/0.96/0.9/0.96/0.95/0.86/0.86
	MDP0000257129	1,2,3,4,5,6,7,8,9,10,total	0.83/0.9/0.92/0.92/0.96/0.96/0.94/0.98/0.94/0.93/0.84
	MDP0000301666	1,2,3,4,5,6,7,8,9,10,total	0.91/0.9/0.96/0.92/0.98/0.97/0.99/0.96/0.92/0.97/0.91
	MDP0000304113	1,2,3,4,6,8,9,total	0.8/0.89/0.85/0.88/0.83/0.83/0.8/0.81
	MDP0000505247	1,2,3,4,5,6,7,8,9,10,total	0.8/0.91/0.87/0.92/0.88/0.92/0.84/0.93/0.92/0.8/0.81
	MDP0000507805	1,3,4,6,7,total	0.8/0.81/0.83/0.8/0.8/0.8
	MDP0000514115	1,2,3,4,5,6,7,8,9,total	0.81/0.86/0.85/0.89/0.81/0.84/0.81/0.83/0.83/0.82
	MDP0000767097	1,total	0.8/0.8
	MDP0000794439	1,2,3,4,5,6,8,9,total	0.87/0.92/0.86/0.9/0.82/0.89/0.84/0.9/0.88
	MDP0000935652	1,2,3,4,5,6,7,8,9,10,total	0.9/0.96/0.95/0.97/0.94/0.97/0.93/0.96/0.95/0.89/0.91
	MDP0000935996	1,2,3,4,5,6,7,8,9,10,total	0.95/0.97/0.95/0.96/0.89/0.95/0.87/0.89/0.93/0.8/0.95
bZIP	MDP0000250947	1,2,3,4,6,8,9,total	-0.87/-0.89/-0.87/-0.9/-0.85 /-0.8/-0.83/-0.88
	MDP0000279891	1,2,3,4,6,9,total	-0.82/-0.86/-0.81/-0.84/-0.82/-0.83/-0.82
	MDP0000280559	1,2,3,4,total	0.9/0.86/0.83/0.81/0.9
	MDP0000310001	1,2,3,4,5,6,7,8,10,total	0.86/0.86/0.89/0.87/0.81/0.84/0.86/0.84/0.8/0.86
	MDP0000680042	1,2,4,total	-0.84/-0.81/-0.8/-0.84
	MDP0000738631	1,2,total	-0.83/-0.82/-0.83
	MDP0000834642	1,2,3,4,6,8,total	-0.83/-0.87/-0.86/-0.84/-0.82/-0.81 /-0.83
Aux/IAA	MDP0000174664	1,total	-0.85/-0.84
	MDP0000176753	1,2,3,4,6,8,9,total	-0.86/-0.91/-0.88/-0.87/-0.85/-0.82 -0.81/-0.87
	MDP0000223496	1,2,3,4,5,6,7,8,9,10,total	0.81/0.82/0.89/0.86/0.96/0.92/0.96/0.94/0.89/0.97/0.81
	MDP0000296324	1,total	-0.8/-0.8
	MDP0000580010	1,2,total	-0.83/-0.81/-0.83
B3	MDP0000125949	1,2,3,total	-0.86/-0.84/-0.8/-0.86
	MDP0000192438	1,total	-0.84/-0.83
Global	MDP0000225062	1,2,total	-0.87/-0.82/-0.87
	MDP0000913105	1,2,3,total	-0.89/-0.85/-0.82/-0.89
Histone	MDP0000127989	1,2,3,total	-0.9/-0.85/-0.83/-0.89

	MDP0000322510	1,2,3,4,total	0.82/0.83/0.83/0.85/0.82
JUMONJI	MDP0000162827	1,3,total	-0.86/-0.82/-0.85
	MDP0000201290	1,2,4,total	-0.81/-0.83/-0.81/-0.82
	MDP0000298212	1,2,3,4,6,9,total	-0.85/-0.89/-0.83/-0.85/-0.86/-0.87/-0.85
PHOR1	MDP0000129445	1,2,3,4,5,6,7,8,9,10,total	0.92/0.93/0.97/0.95/0.94/0.96/0.96/0.95/0.9/0.92/0.93
	MDP0000199588	1,2,3,4,5,6,7,8,9,10,total	0.81/0.85/0.9/0.89/0.98/0.94/0.97/0.97/0.92/0.98/0.81
	MDP0000391355	1,2,3,4,5,6,7,8,10,total	0.9/0.83/0.9/0.85/0.85/0.86 0.88/0.83/0.82/0.9
	MDP0000928620	1,2,3,4,5,6,7,8,9,10,total	0.95/0.96/0.97/0.96/0.9/0.95/0.92/0.92/0.89/0.85/0.95
Psudo ARR	MDP0000150823	1,total	-0.81/-0.81
	MDP0000917419	1,2,3,4,6,7,total	0.9/0.82/0.88/0.81/0.8/0.83/0.9
SET-domain	MDP0000170814	1,total	-0.85/-0.85
	MDP0000248927	1,2,3,4,6,9,total	0.8/0.88/0.8/0.85/0.8/0.81/0.8
SNF7	MDP0000292973	1,2,3,6,9,total	0.8/0.81/0.8/0.83/0.84/0.81
	MDP0000304929	1,2,3,4,5,6,7,8,total	0.82/0.82/0.84/0.81/0.8/0.83/0.81/0.81/0.82
	MDP0000716576	1,2,3,4,5,6,7,8,9,10,total	0.81/0.87/0.85/0.88/0.9/0.92/0.85/0.9/0.94/0.82/0.82
OFP	MDP0000119191	1,2,total	-0.81/-0.84/-0.82
	MDP0000156202	1,2,total	-0.85/-0.84/-0.85

Note: Anthocyanin category No. refer to table 2
